# Supplementary material for: New Insights into Renal Failure in a Cohort of 317 Patients with Autosomal Dominant Forms of Alport Syndrome: Report of Two Novel Heterozygous Mutations in COL4A3
Source: J Clin Med. 2022 Aug 19;11(16):4883. doi: 10.3390/jcm11164883 (PMC9409901; doi:10.3390/jcm11164883)
Supplement: Supplementary file 1 [file jcm-11-04883-s001.zip › jcm-1756647-supplementary.pdf]

Patient	Sex	Gene	Chromosome position	cDNA	Protein	Age at diagnostic	Current age	Age at renal insufficiency	Renal Event	Age at onset	Hematuria	Microhematuria	Proteinuria	Hypoacusia	Reference
1	Mujer	COL4A4	NC_000002.11:g.227954583C>T	NM_000092.4:c.1459+1G>A		35	35			6	Sí	-	Sí	-	[30]
2	Mujer	COL4A4	NC_000002.11:g.227954583C>T	NM_000092.4:c.1459+1G>A		<42	42			42	Sí	-	Sí	-	[30]
3	Mujer	COL4A4	NC_000002.11:g.227954583C>T	NM_000092.4:c.1459+1G>A		<69	69			69	Sí	-	Sí	-	[30]
4	Hombre	COL4A3	NC_000002.11:g.228175583G>A	NM_000091.4:c.4847G>A	NP_000082.2:p.Cys1616Tyr	41	46	46	ESRD	41	Sí	-	Sí	-	[28]
5	Hombre	COL4A3	NC_000002.11:g.228175583G>A	NM_000091.4:c.4847G>A	NP_000082.2:p.Cys1616Tyr	37	55	55	ESRD	37	-	-	Sí	-	[28]
6	Hombre	COL4A3	NC_000002.11:g.228175583G>A	NM_000091.4:c.4847G>A	NP_000082.2:p.Cys1616Tyr	49	50	50	ESRD	49	-	-	Sí	-	[28]
7	Mujer	COL4A3	NC_000002.11:g.228175583G>A	NM_000091.4:c.4847G>A	NP_000082.2:p.Cys1616Tyr	22	22			22	-	-	Sí	-	[28]
8	Mujer	COL4A3	NC_000002.11:g.228145635G>A	NM_000091.4:c.2401G>A	NP_000082.2:p.Gly801Arg	26	52	52	ESRD	26	-	-	Sí	-	[28]
9	Mujer	COL4A3	NC_000002.11:g.228145635G>A	NM_000091.4:c.2401G>A	NP_000082.2:p.Gly801Arg	62	62	62	ESRD	62	-	-	Sí	-	[28]
10	Mujer	COL4A3	NC_000002.11:g.228145635G>A	NM_000091.4:c.2401G>A	NP_000082.2:p.Gly801Arg	54	59			54	-	-	Sí	-	[28]
11	Mujer	COL4A3	NC_000002.11:g.228145635G>A	NM_000091.4:c.2401G>A	NP_000082.2:p.Gly801Arg	54	58			54	-	-	Sí	-	[28]
12	Mujer	COL4A3	NC_000002.11:g.228145635G>A	NM_000091.4:c.2401G>A	NP_000082.2:p.Gly801Arg	25	25			25	Sí	-	Sí	-	[28]
13	Mujer	COL4A3	NC_000002.11:g.228145635G>A	NM_000091.4:c.2401G>A	NP_000082.2:p.Gly801Arg	28	28			28	Sí	-	Sí	-	[28]
14	Mujer	COL4A3	NC_000002.11:g.228157923C>T	NM_000091.4:c.3227C>T	NP_000082.2:p.Pro1076Leu	22	22			22	-	-	Sí	Sí	[34]
15	Hombre	COL4A3	NC_000002.11:g.228157923C>T	NM_000091.4:c.3227C>T	NP_000082.2:p.Pro1076Leu	16	16			16	-	-	-	Sí	[34]
16	Hombre	COL4A3	NC_000002.11:g.228157923C>T	NM_000091.4:c.3227C>T	NP_000082.2:p.Pro1076Leu	21	21			21	-	-	Sí	Sí	[34]
17	Mujer	COL4A3	NC_000002.11:g.228157923C>T	NM_000091.4:c.3227C>T	NP_000082.2:p.Pro1076Leu	20	20			20	-	-	Sí	Sí	[34]
18	Mujer	COL4A3	NC_000002.11:g.228157923C>T	NM_000091.4:c.3227C>T	NP_000082.2:p.Pro1076Leu	47	47			47	-	-	Sí	Sí	[34]
19	Hombre	COL4A3	NC_000002.11:g.228122329G>A	NM_000091.4:c.998G>A	NP_000082.2:p.Gly333Glu	26	26			26	Sí	-	-	Sí	[34]
20	Hombre	COL4A3	NC_000002.11:g.228122329G>A	NM_000091.4:c.998G>A	NP_000082.2:p.Gly333Glu	25	25			25	Sí	-	-	-	[34]
21	Hombre	COL4A3	NC_000002.11:g.228122329G>A	NM_000091.4:c.998G>A	NP_000082.2:p.Gly333Glu	27	27			27	Sí	-	-	-	[34]
22	Mujer	COL4A3	NC_000002.11:g.228122329G>A	NM_000091.4:c.998G>A	NP_000082.2:p.Gly333Glu	27	27			27	Sí	-	-	-	[34]
23	Mujer	COL4A3	NC_000002.11:g.228122329G>A	NM_000091.4:c.998G>A	NP_000082.2:p.Gly333Glu	53	53			53	Sí	-	-	Sí	[34]
24	Mujer	COL4A3	NC_000002.11:g.228122329G>A	NM_000091.4:c.998G>A	NP_000082.2:p.Gly333Glu	56	56	56	CKD	56	Sí	-	Sí	Sí	[34]
25	Hombre	COL4A3	NC_000002.11:g.228122329G>A	NM_000091.4:c.998G>A	NP_000082.2:p.Gly333Glu	47	47			47	Sí	-	-	-	[34]
26	Hombre	COL4A3	NC_000002.11:g.228122329G>A	NM_000091.4:c.998G>A	NP_000082.2:p.Gly333Glu	54	54			54	Sí	-	Sí	Sí	[34]
27	Hombre	COL4A3	NC_000002.11:g.228122329G>A	NM_000091.4:c.998G>A	NP_000082.2:p.Gly333Glu	62	62			62	Sí	-	-	-	[34]
28	Hombre	COL4A3	NC_000002.11:g.228122329G>A	NM_000091.4:c.998G>A	NP_000082.2:p.Gly333Glu	58	58			58	Sí	-	Sí	Sí	[34]
29	Hombre	COL4A4	NC_000002.11:g.227914787delT	NM_000092.4:c.3213delA	NP_000083.3:p.Gly1072Glufs*69	31	37			37	-	Sí	No	-	[32]
30	Hombre	COL4A4	NC_000002.11:g.227914787delT	NM_000092.4:c.3213delA	NP_000083.3:p.Gly1072Glufs*69	32	34			34	-	Sí	No	-	[32]
31	Hombre	COL4A4	NC_000002.11:g.227914787delT	NM_000092.4:c.3213delA	NP_000083.3:p.Gly1072Glufs*69	24	32			32	-	Sí	No	-	[32]
32	Mujer	COL4A4	NC_000002.11:g.227914787delT	NM_000092.4:c.3213delA	NP_000083.3:p.Gly1072Glufs*69	26	27			27	-	Sí	No	-	[32]
33	Mujer	COL4A4	NC_000002.11:g.227872944A>C	NM_000092.4:c.4599T>G	NP_000083.3:p.Tyr1533*	46	46			46	-	Sí	Sí	-	[37]
34	Hombre	COL4A4	NC_000002.11:g.227872944A>C	NM_000092.4:c.4599T>G	NP_000083.3:p.Tyr1533*	52	52			52	Sí	-	No	-	[37]
35	Mujer	COL4A4	NC_000002.11:g.227872944A>C	NM_000092.4:c.4599T>G	NP_000083.3:p.Tyr1533*	59	59			59	Sí	-	No	-	[37]
36	Mujer	COL4A4	NC_000002.11:g.227942760C>T	NM_000092.4:c.1837G>A	NP_000083.3:p.Gly613Arg	7	7			7	-	Sí	-	-	[7]
37	Hombre	COL4A4	NC_000002.11:g.227942760C>T	NM_000092.4:c.1837G>A	NP_000083.3:p.Gly613Arg	45	45			45	-	Sí	-	-	[7]
38	Mujer	COL4A4	NC_000002.11:g.227942760C>T	NM_000092.4:c.1837G>A	NP_000083.3:p.Gly613Arg	41	41			41	-	Sí	-	-	[7]
39	Mujer	COL4A4	NC_000002.11:g.227966616C>A	NM_000092.4:c.940G>T	NP_000083.3:p.Gly314Cys	7	7			7	-	Sí	-	-	[7]
40	Hombre	COL4A4	NC_000002.11:g.227966616C>A	NM_000092.4:c.940G>T	NP_000083.3:p.Gly314Cys	48	48			48	-	Sí	Sí	-	[7]

41	Hombre	COL4A4	NC_000002.11:g.227966616C>A	NM_000092.4:c.940G>T	NP_000083.3:p.Gly314Cys	unknown	unknown		ESRD	unknown	-	-	-	-	[7]
42	Mujer	COL4A4	NC_000002.11:g.227953413C>A	NM_000092.4:c.1579G>T	NP_000083.3:p.Gly527Cys	26	26			26	Sí	Sí	Sí	-	[7]
43	Hombre	COL4A4	NC_000002.11:g.227953413C>A	NM_000092.4:c.1579G>T	NP_000083.3:p.Gly527Cys	40	40			40	-	Sí	-	-	[7]
44	Mujer	COL4A4	NC_000002.11:g.227953413C>A	NM_000092.4:c.1579G>T	NP_000083.3:p.Gly527Cys	47	47			47	-	Sí	-	-	[7]
45	Hombre	COL4A4	NC_000002.11:g.227953413C>A	NM_000092.4:c.1579G>T	NP_000083.3:p.Gly527Cys	76	76			76	-	Sí	-	-	[7]
46	Mujer	COL4A4	NC_000002.11:g.227953413C>A	NM_000092.4:c.1579G>T	NP_000083.3:p.Gly527Cys	21	21			21	Sí	Sí	-	-	[7]
47	Hombre	COL4A4	NC_000002.11:g.227953413C>A	NM_000092.4:c.1579G>T	NP_000083.3:p.Gly527Cys	11	11			11	-	Sí	-	-	[7]
48	Hombre	COL4A4	NC_000002.11:g.227924119A>C	NM_000092.4:c.2383+2T>G		20	20			20	-	Sí	-	-	[7]
49	Mujer	COL4A4	NC_000002.11:g.227924119A>C	NM_000092.4:c.2383+2T>G		11	11			11	-	Sí	-	-	[7]
50	Hombre	COL4A4	NC_000002.11:g.227924119A>C	NM_000092.4:c.2383+2T>G		49	49		CKD	49	-	Sí	Sí	-	[7]
51	Hombre	COL4A4	NC_000002.11:g.227924119A>C	NM_000092.4:c.2383+2T>G		73	73	60	ESRD	73	-	Sí	Sí	-	[7]
52	Mujer	COL4A4	NC_000002.11:g.227912190C>T	NM_000092.4:c.3289+1G>A		16	16			16	-	Sí	Sí	-	[7]
53	Mujer	COL4A4	NC_000002.11:g.227912190C>T	NM_000092.4:c.3289+1G>A		11	11			11	-	Sí	-	-	[7]
54	Hombre	COL4A4	NC_000002.11:g.227912190C>T	NM_000092.4:c.3289+1G>A		9	9			9	-	Sí	-	-	[7]
55	Mujer	COL4A4	NC_000002.11:g.227912190C>T	NM_000092.4:c.3289+1G>A		6	6			6	-	Sí	-	-	[7]
56	Hombre	COL4A4	NC_000002.11:g.227912190C>T	NM_000092.4:c.3289+1G>A		34	34		CKD	34	-	Sí	Sí	-	[7]
57	Mujer	COL4A4	NC_000002.11:g.227912190C>T	NM_000092.4:c.3289+1G>A		40	40			40	-	Sí	Sí	-	[7]
58	Hombre	COL4A4	NC_000002.11:g.227912190C>T	NM_000092.4:c.3289+1G>A		42	42			42	-	Sí	-	-	[7]
59	Hombre	COL4A4	NC_000002.11:g.227912190C>T	NM_000092.4:c.3289+1G>A		55	55	45	ESRD	55	-	Sí	Sí	-	[7]
60	Mujer	COL4A4	NC_000002.11:g.227912190C>T	NM_000092.4:c.3289+1G>A		64	64	60	ESRD	64	-	Sí	Sí	-	[7]
61	Mujer	COL4A4	NC_000002.11:g.227942712delG	NM_000092.4:c.1889delC	NP_000083.3:p.Pro630Glnfs*23	25	25			25	-	Sí	Sí	-	[7]
62	Hombre	COL4A4	NC_000002.11:g.227942712delG	NM_000092.4:c.1889delC	NP_000083.3:p.Pro630Glnfs*23	55	55			25	-	Sí	-	-	[7]
63	Hombre	COL4A4	NC_000002.11:g.227953413delC	NM_000092.4:c.1580delG	NP_000083.3:p.Gly527Valfs*126	43	43	40	CKD	43	-	Sí	Sí	-	[7]
64	Mujer	COL4A4	NC_000002.11:g.227953413delC	NM_000092.4:c.1580delG	NP_000083.3:p.Gly527Valfs*126	15	15			15	-	Sí	Sí	-	[7]
65	Hombre	COL4A4	NC_000002.11:g.227875059delC	NM_000092.4:c.4494delG	NP_000083.3:p.Gln1499Lysfs*53	33	33			33	-	Sí	Sí	-	[7]
66	Mujer	COL4A4	NC_000002.11:g.227966581C>G	NM_000092.4:c.975G>C	NP_000083.3:p.Lys325Asn	8	31	29	ESRD	8	Sí	-	-	Sí	[19]
67	Hombre	COL4A4	NC_000002.11:g.227966581C>G	NM_000092.4:c.975G>C	NP_000083.3:p.Lys325Asn	5	26			5	Sí	-	-	-	[19]
68	Hombre	COL4A4	NC_000002.11:g.227966581C>G	NM_000092.4:c.975G>C	NP_000083.3:p.Lys325Asn	40	66	66	ESRD	40	Sí	-	-	Sí	[19]
69	Mujer	COL4A4	NC_000002.11:g.227966581C>G	NM_000092.4:c.975G>C	NP_000083.3:p.Lys325Asn	40	54			40	Sí	-	-	Sí	[19]
70	Hombre	COL4A4	NC_000002.11:g.227966581C>G	NM_000092.4:c.975G>C	NP_000083.3:p.Lys325Asn	54	66	60	Dialysis	54	-	-	-	Sí	[19]
71	Mujer	COL4A4	NC_000002.11:g.227886785T>A	NM_000092.4:c.4195A>T	NP_000083.3:p.Met1399Leu	26	26			26	Sí	-	Sí	Sí	[33]
72	Hombre	COL4A4	NC_000002.11:g.227886785T>A	NM_000092.4:c.4195A>T	NP_000083.3:p.Met1399Leu	60	62	60	Dialysis	60	-	-	-	-	[33]
73	Hombre	COL4A4	NC_000002.11:g.227886785T>A	NM_000092.4:c.4195A>T	NP_000083.3:p.Met1399Leu	45	45	62	Renal failure	45	Sí	-	Sí	Sí	[33]
74	Hombre	COL4A4	NC_000002.11:g.227942800A>G	NM_000092.4:c.1804-7T>C		26	26			26	-	Sí	Sí	Sí	[38]
75	Mujer	COL4A4	NC_000002.11:g.227942800A>G	NM_000092.4:c.1804-7T>C		23	23			23	-	Sí	Sí	Sí	[38]
76	Mujer	COL4A4	NC_000002.11:g.227942800A>G	NM_000092.4:c.1804-7T>C		49	49			49	-	Sí	Sí	Sí	[38]
77	Hombre	COL4A4	NC_000002.11:g.227942800A>G	NM_000092.4:c.1804-7T>C		51	51			51	-	Sí	Sí	-	[38]
78	Hombre	COL4A4	NC_000002.11:g.227914787delT	NM_000092.4:c.3213delA	NP_000083.3:p.Gly1072Glufs*69	31	32			31	-	Sí	No	-	[32]
79	Hombre	COL4A4	NC_000002.11:g.227914787delT	NM_000092.4:c.3213delA	NP_000083.3:p.Gly1072Glufs*69	45	75	75	CKD	45	-	Sí	Sí	-	[32]
80	Mujer	COL4A4	NC_000002.11:g.227914787delT	NM_000092.4:c.3213delA	NP_000083.3:p.Gly1072Glufs*69	40	65		CKD	65	-	Sí	Sí	-	[32]
81	Hombre	COL4A4	NC_000002.11:g.227914787delT	NM_000092.4:c.3213delA	NP_000083.3:p.Gly1072Glufs*69	45	61	60	ESRD	45	-	Sí	Sí	-	[32]
82	Hombre	COL4A4	NC_000002.11:g.227914787delT	NM_000092.4:c.3213delA	NP_000083.3:p.Gly1072Glufs*69	43	58	56	ESRD	43	-	Sí	Sí	-	[32]

83	Mujer	COL4A4	NC_000002.11:g.227914787delT	NM_000092.4:c.3213delA	NP_000083.3:p.Gly1072Glufs*69	35	41			35	-	Sí	No	-	[32]
84	Mujer	COL4A4	NC_000002.11:g.227917119C>A	NM_000092.4:c.2870G>T	NP_000083.3:p.Gly957Val	13	31			13	-	Sí	-	-	[22]
85	Hombre	COL4A4	NC_000002.11:g.227917119C>A	NM_000092.4:c.2870G>T	NP_000083.3:p.Gly957Val	<52	52	52	RT	52	-	-	-	-	[22]
86	Hombre	COL4A4	NC_000002.11:g.227917119C>A	NM_000092.4:c.2870G>T	NP_000083.3:p.Gly957Val	1	1			<1	-	Sí	-	-	[22]
87	Hombre	COL4A4	NC_000002.11:g.227917119C>A	NM_000092.4:c.2870G>T	NP_000083.3:p.Gly957Val	<59	59			59	-	Sí	-	-	[22]
88	Mujer	COL4A4	NC_000002.11:g.227917119C>A	NM_000092.4:c.2870G>T	NP_000083.3:p.Gly957Val	<41	41	<41	CKD	41	-	-	-	-	[22]
89	Hombre	COL4A4	NC_000002.11:g.227872214A>T	NM_000092.4:c.4900T>A	NP_000083.3:p.Cys1634Ser	37	45			37	Sí	Sí	-	Sí	[22]
90	Hombre	COL4A4	NC_000002.11:g.227872214A>T	NM_000092.4:c.4900T>A	NP_000083.3:p.Cys1634Ser	3	13			13	Sí	Sí	-	-	[22]
91	Hombre	COL4A4	NC_000002.11:g.227872214A>T	NM_000092.4:c.4900T>A	NP_000083.3:p.Cys1634Ser	<51	51			51	Sí	Sí	-	Sí	[22]
92	Hombre	COL4A3	NC_000002.11:g.228155526G>T	NM_000091.4:c.3134G>T	NP_000082.2:p.Gly1045Val	18	55	55	CKD	18	-	Sí	Sí	Sí	[22]
93	Mujer	COL4A3	NC_000002.11:g.228155526G>T	NM_000091.4:c.3134G>T	NP_000082.2:p.Gly1045Val	32	32			32	-	Sí	-	-	[22]
94	Hombre	COL4A3	NC_000002.11:g.228155526G>T	NM_000091.4:c.3134G>T	NP_000082.2:p.Gly1045Val	24	24			24	-	Sí	-	-	[22]
95	Hombre	COL4A3	NC_000002.11:g.228155526G>T	NM_000091.4:c.3134G>T	NP_000082.2:p.Gly1045Val	21	21			21	-	Sí	-	-	[22]
96	Mujer	COL4A3	NC_000002.11:g.228155526G>T	NM_000091.4:c.3134G>T	NP_000082.2:p.Gly1045Val	68	68	68	CKD	<68	-	Sí	-	-	[22]
97	Hombre	COL4A3	NC_000002.11:g.228155526G>T	NM_000091.4:c.3134G>T	NP_000082.2:p.Gly1045Val	44	44			44	-	Sí	-	-	[22]
98	Hombre	COL4A3	NC_000002.11:g.228110697G>A	NM_000091.4:c.352G>A	NP_000082.2:p.Gly118Arg	29	29			29	-	-	Sí	-	[28]
99	Mujer	COL4A3	NC_000002.11:g.228110697G>A	NM_000091.4:c.352G>A	NP_000082.2:p.Gly118Arg	70	70			70	-	-	Sí	-	[28]
100	Hombre	COL4A3	NC_000002.11:g.228154724G>A	NM_000091.4:c.2990G>A	NP_000082.2:p.Gly997Glu	30	38			30	Sí	-	Sí	-	[28]
101	Hombre	COL4A3	NC_000002.11:g.228154724G>A	NM_000091.4:c.2990G>A	NP_000082.2:p.Gly997Glu	15	15			15	-	-	Sí	-	[28]
102	Mujer	COL4A3	NC_000002.11:g.228154724G>A	NM_000091.4:c.2990G>A	NP_000082.2:p.Gly997Glu	56	70			56	Sí	-	Sí	-	[28]
103	Mujer	COL4A3	NC_000002.11:g.228154724G>A	NM_000091.4:c.2990G>A	NP_000082.2:p.Gly997Glu	30				30	Sí	-	Sí	-	[28]
104	Mujer	COL4A3	NC_000002.11:g.228144593T>A	NM_000091.4:c.2210T>A	NP_000082.2:p.Leu737His	19	35			19	-	-	Sí	-	[28]
105	Mujer	COL4A3	NC_000002.11:g.228144593T>A	NM_000091.4:c.2210T>A	NP_000082.2:p.Leu737His	57	58			58	-	-	Sí	-	[31]
106	Mujer	COL4A4	NC_000002.11:g.227963516T>G	NM_000092.4:c.1100-2A>C		12				12	-	Sí	-	-	[31]
107	Hombre	COL4A4	NC_000002.11:g.227963516T>G	NM_000092.4:c.1100-2A>C		5				5	Sí	-	-	-	[31]
108	Mujer	COL4A4	NC_000002.11:g.227963516T>G	NM_000092.4:c.1100-2A>C		38				38	Sí	-	-	-	[31]
109	Hombre	COL4A4	NC_000002.11:g.227915693_227915698delCTGGTCinsG	NM_000092.4:c.3145_3150delGACCAGinsC	NP_000083.3:p.Asp1049Argfs*2	32	32			32	Sí	-	-	-	[23]
110	Hombre	COL4A4	NC_000002.11:g.227915693_227915698delCTGGTCinsG	NM_000092.4:c.3145_3150delGACCAGinsC	NP_000083.3:p.Asp1049Argfs*2	7	7			7	Sí	-	-	-	[23]
111	Mujer	COL4A4	NC_000002.11:g.227919308A>C	NM_000092.4:c.2860+2T>G		41	48			41	Sí	-	-	-	[23]
112	Hombre	COL4A4	NC_000002.11:g.227919308A>C	NM_000092.4:c.2860+2T>G		18	18			18	Sí	-	-	-	[23]
113	Hombre	COL4A4	NC_000002.11:g.227912190C>T	NM_000092.4:c.3289+1G>A		36	36			36	Sí	-	-	-	[23]
114	Mujer	COL4A4	NC_000002.11:g.227912190C>T	NM_000092.4:c.3289+1G>A		13	13			13	Sí	-	-	-	[23]
115	Mujer	COL4A4	NC_000002.11:g.227924184C>G	NM_000092.4:c.2320G>C	NP_000083.3:p.Gly774Arg	38	38			39	Sí	-	-	-	[23]
116	Hombre	COL4A3	NC_000002.11:g.228131759G>T	NM_000091.4:c.1459G>T	NP_000082.2:p.Gly487Cys	38	38			38	Sí	-	-	-	[23]
117	Mujer	COL4A3	NC_000002.11:g.228154778G>A	NM_000091.4:c.3044G>A	NP_000082.2:p.Gly1015Glu	36	36			36	Sí	-	-	-	[23]
118	Mujer	COL4A4	NC_000002.11:g.227924184C>G	NM_000092.4:c.2320G>C	NP_000083.3:p.Gly774Arg	36	36			36	Sí	-	-	-	[23]
119	Hombre	COL4A4	NC_000002.11:g.227924184C>G	NM_000092.4:c.2320G>C	NP_000083.3:p.Gly774Arg	18	18			18	Sí	-	-	-	[23]
120	Hombre	COL4A4	NC_000002.11:g.227924184C>G	NM_000092.4:c.2320G>C	NP_000083.3:p.Gly774Arg	16	16			16	Sí	-	-	-	[23]
121	Mujer	COL4A3	NC_000002.11:g.228131759G>T	NM_000091.4:c.1459G>T	NP_000082.2:p.Gly487Cys	13	13			13	Sí	-	-	-	[23]
122	Mujer	COL4A3	NC_000002.11:g.228154778G>A	NM_000091.4:c.3044G>A	NP_000082.2:p.Gly1015Glu	20	20			20	Sí	-	-	-	[23]
123	Mujer	COL4A3	NC_000002.11:g.228154778G>A	NM_000091.4:c.3044G>A	NP_000082.2:p.Gly1015Glu	16	16			16	Sí	-	-	-	[23]
124	Mujer	COL4A3	NC_000002.11:g.228160015_228160016insAGG	NM_000091.4:c.3548_3549insAGG	NP_000082.2:p.Gly1183_Asn1184insGly	56	56			56	Sí	-	-	-	[23]

125	Hombre	COL4A3	NC_000002.11:g.228128660G>A	NM_000091.4:c.1315G>A	NP_000082.2:p.Gly439Ser	36	36	35	ESRD	<36	Sí	-	Sí	Sí	[18]
126	Mujer	COL4A4	NC_000002.11:g.227958874_227958891delCTCCAGGCAAGCCAGGTG	NM_000092.4:c.1323_1340delTGGCTTGCCTGGAGCACC	NP_000083.3:p.Gly442_Pro447del	17	45			17	-	-	Sí	-	[2]
127	Hombre	COL4A3	NC_000002.11:g.228153966T>A	NM_000091.4:c.2980+2T>A		42	43			42	-	-	Sí	-	[2]
128	Mujer	COL4A4	NC_000002.11:g.227942789T>C	NM_000092.4:c.1808A>G	NP_000083.3:p.Asp603Gly	35	46			42	-	-	Sí	-	[2]
129	Mujer	COL4A4	NC_000002.11:g.227945229C>A	NM_000092.4:c.1733G>T	NP_000083.3:p.Gly578Val	9	48			9	-	-	Sí	-	[2]
130	Hombre	COL4A4	NC_000002.11:g.227945229C>A	NM_000092.4:c.1733G>T	NP_000083.3:p.Gly578Val	32	82	63	ESRD	32	-	-	Sí	-	[2]
131	Mujer	COL4A4	NC_000002.11:g.227919444C>T	NM_000092.4:c.2726G>A	NP_000083.3:p.Gly909Glu	16	26			16	-	-	Sí	-	[2]
132	Hombre	COL4A3	NC_000002.11:g.228137761G>A	NM_000091.4:c.1855G>A	NP_000082.2:p.Gly619Arg	5	26			5	-	-	Sí	-	[2]
133	Mujer	COL4A3	NC_000002.11:g.228159760G>A	NM_000091.4:c.3499G>A	NP_000082.2:p.Gly1167Arg	5	31			5	-	-	Sí	-	[2]
134	Hombre	COL4A3	NC_000002.11:g.228137807G>A	NM_000091.4:c.1901G>A	NP_000082.2:p.Gly634Glu	11	36			11	-	-	Sí	-	[2]
135	Hombre	COL4A3	NC_000002.11:g.228137761G>A	NM_000091.4:c.1855G>A	NP_000082.2:p.Gly619Arg	20	45			20	-	-	Sí	-	[2]
136	Hombre	COL4A4	NC_000002.11:g.227958874_227958891delCTCCAGGCAAGCCAGGTG	NM_000092.4:c.1323_1340delTGGCTTGCCTGGAGCACC	NP_000083.3:p.Gly442_Pro447del	17	29			17	-	-	Sí	-	[2]
137	Mujer	COL4A4	NC_000002.11:g.227924932C>T	NM_000092.4:c.2084G>A	NP_000083.3:p.Gly695Asp	6	11			6	-	-	Sí	-	[2]
138	Mujer	COL4A3	NC_000002.11:g.228159725G>A	NM_000091.4:c.3464G>A	NP_000082.2:p.Gly1155Asp	44	69			44	-	-	Sí	Sí	[2]
139	Hombre	COL4A3	NC_000002.11:g.228155526G>T	NM_000091.4:c.3134G>T	NP_000082.2:p.Gly1045Val	37	37			37	-	Sí	-	-	[22]
140	Mujer	COL4A3	NC_000002.11:g.228155526G>T	NM_000091.4:c.3134G>T	NP_000082.2:p.Gly1045Val	84	88	84	Dialysis	84	-	-	-	Sí	[22]
141	Hombre	COL4A3	NC_000002.11:g.228162479G>T	NM_000091.4:c.3655G>T	NP_000082.2:p.Gly1219Cys	18	49	45	Dialysis	18	-	Sí	Sí	-	[22]
142	Hombre	COL4A3	NC_000002.11:g.228162479G>T	NM_000091.4:c.3655G>T	NP_000082.2:p.Gly1219Cys	20	46			20	-	Sí	-	Sí	[22]
143	Hombre	COL4A3	NC_000002.11:g.228162479G>T	NM_000091.4:c.3655G>T	NP_000082.2:p.Gly1219Cys	45	45			<45	-	Sí	Sí	Sí	[22]
144	Hombre	COL4A3	NC_000002.11:g.228162479G>T	NM_000091.4:c.3655G>T	NP_000082.2:p.Gly1219Cys	20	36			20	-	Sí	-	-	[22]
145	Mujer	COL4A3	NC_000002.11:g.228162479G>T	NM_000091.4:c.3655G>T	NP_000082.2:p.Gly1219Cys	20	20			20	-	Sí	-	-	[22]
146	Mujer	COL4A3	NC_000002.11:g.228162479G>T	NM_000091.4:c.3655G>T	NP_000082.2:p.Gly1219Cys	<70	72	70	Dialysis	70	-	-	-	-	[22]
147	Hombre	COL4A3	NC_000002.11:g.228172594T>C	NM_000091.4:c.4421T>C	NP_000082.2:p.Leu1474Pro	36	40	40	ESRD	36	-	-	-	-	[5]
148	Hombre	COL4A3	NC_000002.11:g.228172594T>C	NM_000091.4:c.4421T>C	NP_000082.2:p.Leu1474Pro	36	40	40	ESRD	36	-	-	-	-	[5]
149	Mujer	COL4A3	NC_000002.11:g.228112275G>T	NM_000091.4:c.443G>T	NP_000082.2:p.Gly148Val	35	35			<35	Sí	-	Sí	-	[5]
150	Hombre	COL4A3	NC_000002.11:g.228112275G>T	NM_000091.4:c.443G>T	NP_000082.2:p.Gly148Val	65	72	82	ESRD	65	-	-	-	-	[5]
151	Hombre	COL4A3	NC_000002.11:g.228176554C>T	NM_000091.4:c.4981C>T	NP_000082.2:p.Arg1661Cys	18	37	37	ESRD	18	Sí	-	Sí	-	[5]
152	Hombre	COL4A3	NC_000002.11:g.228176554C>T	NM_000091.4:c.4981C>T	NP_000082.2:p.Arg1661Cys	33	33			33	-	-	Sí	-	[5]
153	Mujer	COL4A3	NC_000002.11:g.228142227G>A	NM_000091.4:c.2083G>A	NP_000082.2:p.Gly695Arg	36	50	50	ESRD	36	-	-	Sí	Sí	[5]
154	Mujer	COL4A3	NC_000002.11:g.228142227G>A	NM_000091.4:c.2083G>A	NP_000082.2:p.Gly695Arg	64	64			<64	-	-	Sí	Sí	[5]
155	Hombre	COL4A3	NC_000002.11:g.228142227G>A	NM_000091.4:c.2083G>A	NP_000082.2:p.Gly695Arg	39	39			39	Sí	-	Sí	Sí	[5]
156	Mujer	COL4A3	NC_000002.11:g.228142227G>A	NM_000091.4:c.2083G>A	NP_000082.2:p.Gly695Arg	37	37			37	-	-	Sí	Sí	[5]
157	Hombre	COL4A3	NC_000002.11:g.228142227G>A	NM_000091.4:c.2083G>A	NP_000082.2:p.Gly695Arg	40	40			40	Sí	-	No	Sí	[5]
158	Mujer	COL4A4	NC_000002.11:g.227983440C>T	NM_000092.4:c.410G>A	NP_000083.3:p.Gly137Asp	35	35			35	Sí	-	Sí	-	[5]
159	Hombre	COL4A4	NC_000002.11:g.227983440C>T	NM_000092.4:c.410G>A	NP_000083.3:p.Gly137Asp	32	42	42	ESRD	32	-	-	Sí	-	[5]
160	Mujer	COL4A4	NC_000002.11:g.227983440C>T	NM_000092.4:c.410G>A	NP_000083.3:p.Gly137Asp	32	41	41	ESRD	32	-	-	Sí	-	[5]
161	Hombre	COL4A4	NC_000002.11:g.227983440C>T	NM_000092.4:c.410G>A	NP_000083.3:p.Gly137Asp	44	44	44	ESRD	44	-	-	-	-	[5]
162	Hombre	COL4A4	NC_000002.11:g.227983440C>T	NM_000092.4:c.410G>A	NP_000083.3:p.Gly137Asp	60	<60	60	ESRD	60	-	-	-	-	[5]
163	Mujer	COL4A4	NC_000002.11:g.227917083G>C	NM_000092.4:c.2906C>G	NP_000083.3:p.Ser969*	2	2			2	Sí	-	-	-	[5]
164	Mujer	COL4A4	NC_000002.11:g.227917083G>C	NM_000092.4:c.2906C>G	NP_000083.3:p.Ser969*	5	5			5	Sí	-	-	-	[5]
165	Hombre	COL4A4	NC_000002.11:g.227917083G>C	NM_000092.4:c.2906C>G	NP_000083.3:p.Ser969*	2	2			2	Sí	-	-	-	[5]
166	Hombre	COL4A4	NC_000002.11:g.227917083G>C	NM_000092.4:c.2906C>G	NP_000083.3:p.Ser969*	20	20			20	Sí	-	-	-	[5]

167	Mujer	COL4A4	NC_000002.11:g.227917083G>C	NM_000092.4:c.2906C>G	NP_000083.3:p.Ser969*	35	35			35	Sí	-	Sí	-	[5]
168	Hombre	COL4A3	NC_000002.11:g.228119415G>A	NM_000091.4:c.872G>A	NP_000082.2:p.Gly291Glu	13	13			13	Sí	-	-	-	[25]
169	Hombre	COL4A3	NC_000002.11:g.228119415G>A	NM_000091.4:c.872G>A	NP_000082.2:p.Gly291Glu	22	46			22	-	-	Sí	-	[25]
170	Hombre	COL4A3	NC_000002.11:g.228119415G>A	NM_000091.4:c.872G>A	NP_000082.2:p.Gly291Glu	<45	82	70	ESRD	45	-	-	-	-	[25]
171	Hombre	COL4A4	NC_000002.11:g.227920687C>T	NM_000092.4:c.2690G>A	NP_000083.3:p.Gly897Glu	5	16			5	Sí	-	Sí	-	[15]
172	Hombre	COL4A4	NC_000002.11:g.227920687C>T	NM_000092.4:c.2690G>A	NP_000083.3:p.Gly897Glu	76	76			76	Sí	-	-	-	[15]
173	Mujer	COL4A3	NC_000002.11:g.228159278G>A	NM_000091.4:c.3410G>A	NP_000082.2:p.Gly1137Asp	6	6			6	Sí	-	-	-	[31]
174	Hombre	COL4A3	NC_000002.11:g.228159278G>A	NM_000091.4:c.3410G>A	NP_000082.2:p.Gly1137Asp	38	38			38	Sí	-	-	-	[31]
175	Hombre	COL4A3	NC_000002.11:g.228159278G>A	NM_000091.4:c.3410G>A	NP_000082.2:p.Gly1137Asp	12	12			12	Sí	-	-	-	[31]
176	Hombre	COL4A4	NC_000002.11:g.227927272C>T	NM_000092.4:c.2030G>A	NP_000083.3:p.Gly677Asp	15	20			15	-	Sí	Sí	-	[39]
177	Hombre	COL4A4	NC_000002.11:g.227927272C>T	NM_000092.4:c.2030G>A	NP_000083.3:p.Gly677Asp	16	16			16	-	Sí	Sí	-	[39]
178	Mujer	COL4A4	NC_000002.11:g.227927272C>T	NM_000092.4:c.2030G>A	NP_000083.3:p.Gly677Asp	49	49			49	-	Sí	Sí	-	[39]
179	Mujer	COL4A4	NC_000002.11:g.227927272C>T	NM_000092.4:c.2030G>A	NP_000083.3:p.Gly677Asp	43	43			43	-	Sí	Sí	-	[39]
180	Mujer	COL4A4	NC_000002.11:g.227927272C>T	NM_000092.4:c.2030G>A	NP_000083.3:p.Gly677Asp	72	72			72	-	Sí	Sí	-	[39]
181	Hombre	COL4A3	NC_000002.11:g.228162549G>A	NM_000091.4:c.3725G>A	NP_000082.2:p.Gly1242Asp	21	28	30	ESRD	21	-	Sí	Sí	Sí	[24]
182	Mujer	COL4A3	NC_000002.11:g.228162549G>A	NM_000091.4:c.3725G>A	NP_000082.2:p.Gly1242Asp	25	25			25	Sí	-	Sí	-	[24]
183	Mujer	COL4A3	NC_000002.11:g.228162549G>A	NM_000091.4:c.3725G>A	NP_000082.2:p.Gly1242Asp	38	38			<38	-	-	Sí	-	[24]
184	Hombre	COL4A3	NC_000002.11:g.228162549G>A	NM_000091.4:c.3725G>A	NP_000082.2:p.Gly1242Asp	47	47			47	Sí	-	Sí	-	[24]
185	Mujer	COL4A3	NC_000002.11:g.228162549G>A	NM_000091.4:c.3725G>A	NP_000082.2:p.Gly1242Asp	49	49			49	Sí	-	Sí	-	[24]
186	Hombre	COL4A3	NC_000002.11:g.228162549G>A	NM_000091.4:c.3725G>A	NP_000082.2:p.Gly1242Asp	50	50			50	Sí	-	Sí	-	[24]
187	Hombre	COL4A3	NC_000002.11:g.228162549G>A	NM_000091.4:c.3725G>A	NP_000082.2:p.Gly1242Asp	44	44			44	Sí	-	Sí	-	[24]
188	Mujer	COL4A4	NC_000002.11:g.227920741C>T	NM_000092.4:c.2636G>A	NP_000083.3:p.Gly879Glu	55	63	63	ESRD	55	-	-	-	-	[24]
189	Mujer	COL4A4	NC_000002.11:g.227920741C>T	NM_000092.4:c.2636G>A	NP_000083.3:p.Gly879Glu	30	30			30	-	-	Sí	-	[24]
190	Hombre	COL4A3	NC_000002.11:g.228154724G>A	NM_000091.4:c.2990G>A	NP_000082.2:p.Gly997Glu	35	45	45	CKD	35	-	Sí	Sí	-	[24]
191	Mujer	COL4A3	NC_000002.11:g.228154724G>A	NM_000091.4:c.2990G>A	NP_000082.2:p.Gly997Glu	20	55	55	ESRD	20	-	-	Sí	-	[24]
192	Mujer	COL4A3	NC_000002.11:g.228154724G>A	NM_000091.4:c.2990G>A	NP_000082.2:p.Gly997Glu	unknown	unknown			unknown	-	Sí	Sí	-	[24]
193	Mujer	COL4A3	NC_000002.11:g.228154724G>A	NM_000091.4:c.2990G>A	NP_000082.2:p.Gly997Glu	unknown	unknown			unknown	-	Sí	Sí	-	[24]
194	Mujer	COL4A3	NC_000002.11:g.228110690delG	NM_000091.4:c.345delG	NP_000082.2:p.G115GFSX37	32	48	32	CKD	32	Sí	Desconocido	Sí	Sí	[20]
195	Hombre	COL4A3	NC_000002.11:g.228110690delG	NM_000091.4:c.345delG	NP_000082.2:p.G115GFSX37	6		19	CKD		Sí	Sí	Sí	Sí	[20]
196	Mujer	COL4A3	NC_000002.11:g.228169782G>T	NM_000091.4:c.4235G>T	NP_000082.2:p.G1412V	24	52	31	ESRD	24	Desconocido	Sí	Sí	Sí	[20]
197	Hombre	COL4A3	NC_000002.11:g.228169782G>T	NM_000091.4:c.4235G>T	NP_000082.2:p.G1412V	29	53	34	ESRD	29	Sí	Desconocido	Sí	Desconocido	[20]
198	Hombre	COL4A3	NC_000002.11:g.228169782G>T	NM_000091.4:c.4235G>T	NP_000082.2:p.G1412V	67	83	75	CKD	75	Desconocido	Desconocido	Desconocido	Sí	[20]
199	Mujer	COL4A4	NC_000002.11:g.227876963G>A	NM_000092.4:c.4267C>T	NP_000083.3:p.P1423S	6	49			28	Desconocido	Sí	Sí	Sí	[20]
200	Mujer	COL4A4	NC_000002.11:g.227876963G>A	NM_000092.4:c.4267C>T	NP_000083.3:p.P1423S				CKD		Sí	Desconocido	Sí	Sí	[20]
201	Hombre	COL4A4	NC_000002.11:g.227876963G>A	NM_000092.4:c.4267C>T	NP_000083.3:p.P1423S				ESRD		Sí	Desconocido	Sí	Desconocido	[20]
202	Hombre	COL4A4	NC_000002.11:g.228004876C>G	NM_000092.4: c.192+1G>C		30	55			40	Desconocido	Sí	Sí	Desconocido	[20]
203	Hombre	COL4A4	NC_000002.11:g.228004876C>G	NM_000092.4: c.192+1G>C		75					Sí	Desconocido	Sí	Sí	[20]
204	Hombre	COL4A3	NC_000002.11:g.228029482_228029505del	NM_000091.4:c.40_63del	NP_000082.2:p.Leu14_Leu21del	4	14			4	Desconocido	Sí	Sí	Desconocido	[20]
205	Hombre	COL4A3	NC_000002.11:g.228029482_228029505del	NM_000091.4:c.40_63del	NP_000082.2:p.Leu14_Leu21del		46				Sí	Sí	Desconocido	Sí	[20]
206	Mujer	COL4A3	NC_000002.11:g.228162416G>A	NM_000091.4:c.3592G>A	NP_000082.2:p.Gly1198Ser	28	39				Desconocido	Sí	Sí	Desconocido	[20]
207	Mujer	COL4A3	NC_000002.11:g.228158017_228158025delAAGTCCTGG	NM_000091.4:c.3321_3329delAAGTCCTGG	NP_000082.2:p.Ser1108_Gly1110del	32	32				Desconocido	Sí	Sí	Desconocido	[20]

208	Hombre	COL4A4	NC_000002.11:g.227958868C>T	NM_000092.4:c.1342G>A	NP_000083.3:p.Gly448Ser	28	35	28	Non-progressive CRF		Desconocido	Sí	Sí	Desconocido	[21]
209	Hombre	COL4A4	NC_000002.11:g.227958874_227958891delCTCCAGGCAAGCCAGGTG	NM_000092.4:c.1323_1340delTGGCTTGCCTGGAGCACC	NP_000083.3:p.Gly442_Pro447del	44	66	59	ESRD	27	Sí	Desconocido	Sí	Desconocido	[35]
210	Hombre	COL4A4	NC_000002.11:g.227958874_227958891delCTCCAGGCAAGCCAGGTG	NM_000092.4:c.1323_1340delTGGCTTGCCTGGAGCACC	NP_000083.3:p.Gly442_Pro447del	6	29			6	Sí	Desconocido	Sí	Desconocido	[35]
211	Mujer	COL4A4	NC_000002.11:g.227872267A>C	NM_000092.4:c.4847T>G	NP_000083.3:p.Leu1616Arg	23	67			23	Sí	Desconocido	Sí	Desconocido	[35]
212	Mujer	COL4A4	NC_000002.11:g.227872267A>C	NM_000092.4:c.4847T>G	NP_000083.3:p.Leu1616Arg	15	42			15	Sí	Desconocido	Sí	Desconocido	[35]
213	Hombre	COL4A4	NC_000002.11:g.227967903C>G	NM_000092.4:c.827G>C	NP_000083.3:p.Gly276Ala	31	61	58	ESRD	31	Sí	Desconocido	Sí	Desconocido	[35]
214	Mujer	COL4A4	NC_000002.11:g.227967903C>G	NM_000092.4:c.827G>C	NP_000083.3:p.Gly276Ala	12	30			12	Sí	Desconocido	Sí	Desconocido	[35]
215	Hombre	COL4A4	NC_000002.11:g.227886851G>A	NM_000092.4:c.4129C>T	NP_000083.3:p.Arg1377*	23	54			23	Sí	Desconocido	Sí	Desconocido	[35]
216	Mujer	COL4A4	NC_000002.11:g.227964378C>G	NM_000092.4:c.1057G>C	NP_000083.3:p.Gly353Arg	9	49			9	Sí	Desconocido	Sí	Desconocido	[35]
217	Mujer	COL4A4	NC_000002.11:g.227967903C>G	NM_000092.4:c.827G>C	NP_000083.3:p.Gly276Ala	42	64			42	Sí	Desconocido	Sí	Desconocido	[35]
218	Mujer	COL4A4	NC_000002.11:g.227898125C>T	NM_000092.4:c.3577+1G>A		35	61			35	Sí	Desconocido	Sí	Desconocido	[35]
219	Hombre	COL4A3	NC_000002.11:g.228169754G>A	NM_000091.4:c.4207G>A	NP_000082.2:p.Gly1403Arg	5	33			5	Sí	Desconocido	Sí	Desconocido	[35]
220	Mujer	COL4A3	NC_000002.11:g.228159725G>A	NM_000091.4:c.3464G>A	NP_000082.2:p.Gly1155Asp	44	69			44	Sí	Desconocido	Sí	Sí	[35]
221	Hombre	COL4A3	NC_000002.11:g.228159725G>A	NM_000091.4:c.3464G>A	NP_000082.2:p.Gly1155Asp	13	55			13	Sí	Desconocido	Sí	Desconocido	[35]
222	Hombre	COL4A3	NC_000002.11:g.228149043G>A	NM_000091.4:c.2863G>A	NP_000082.2:p.Gly955Arg	6	50	42	ESRD	6	Sí	Desconocido	Sí	Sí	[35]
223	Mujer	COL4A3	NC_000002.11:g.228109073G>T	NM_000091.4:c.272G>T	NP_000082.2:p.Gly91Val	6				6	Sí	Desconocido	No	Desconocido	[35]
224	Mujer	COL4A3	NC_000002.11:g.228120751G>A	NM_000091.4:c.898G>A	NP_000082.2:p.Gly300Arg	35				35	Sí	Desconocido	Sí	Sí	[35]
225	Hombre	COL4A3	NC_000002.11:g.228128564G>A	NM_000091.4:c.1219G>A	NP_000082.2:p.Gly407Ser	26				26	Sí	Desconocido	Sí	Desconocido	[35]
226	Hombre	COL4A3	NC_000002.11:g.228134680G>A	NM_000091.4:c.1559G>A	NP_000082.2:p.Gly520Asp	4				4	Sí	Desconocido	No	Desconocido	[35]
227	Mujer	COL4A3	NC_000002.11:g.228135597G>A	NM_000091.4:c.1687G>A	NP_000082.2:p.Gly563Arg	25				25	Sí	Desconocido	Desconocido	Sí	[35]
228	Hombre	COL4A3	NC_000002.11:g.228141157G>A	NM_000091.4:c.1984G>A	NP_000082.2:p.Gly662Arg	69			ESRD	69	Sí	Desconocido	Sí	Desconocido	[35]
229	Hombre	COL4A3	NC_000002.11:g.228169782G>A	NM_000091.4:c.4235G>A	NP_000082.2:p.Gly1412Asp	38				38	Sí	Desconocido	Sí	Desconocido	[35]
230	Hombre	COL4A3	NC_000002.11:g.228175623_228175624delCA	NM_000091.4:c.4887_4888delCA	NP_000082.2:p.Tyr1629*	7				7	Sí	Desconocido	Sí	Sí	[35]
231	Mujer	COL4A4	NC_000002.11:g.227946893C>G	NM_000092.4:c.1634G>C	NP_000083.3:p.Gly545Ala	32				32	Sí	Desconocido	Sí	Desconocido	[29]
232	Mujer	COL4A4	NC_000002.11:g.227946893C>G	NM_000092.4:c.1634G>C	NP_000083.3:p.Gly545Ala	23					Desconocido	Desconocido	Desconocido	Sí	[29]
233	Mujer	COL4A4	NC_000002.11:g.227924288_227924289insG	NM_000092.4:c.2219_2220insC	NP_000083.3:p.Val741Cysfs*47	15				15	Sí	Desconocido	Sí	Sí	[29]
234	Hombre	COL4A4	NC_000002.11:g.227920687C>T	NM_000092.4:c.2690G>A	NP_000083.3:p.Gly897Glu	48				48	Sí	Desconocido	Sí	Desconocido	[29]
235	Mujer	COL4A4	NC_000002.11:g.227896736C>A	NM_000092.4:c.3742G>T	NP_000083.3:p.Gly1248*	41		41	ESRD	41	No	Desconocido	Sí	Sí	[29]
236	Mujer	COL4A4	NC_000002.11:g.227872294delG	NM_000092.4:c.4820delC	NP_000083.3:p.Ala1607Valfs*49	4				4	Sí	Desconocido	No	Desconocido	[29]
237	Mujer	COL4A3	NC_000002.11:g.228131783C>T	NM_000091.4:c.1483C>T	NP_000082.2:p.His495Tyr	11				11	Sí	Desconocido	No	Desconocido	[29]
238	Mujer	COL4A3	NC_000002.11:g.228159715G>T	NM_000091.4:c.3454G>T	NP_000082.2:p.Gly1152Cys	10				10	Sí	Desconocido	No	Desconocido	[29]
239	Mujer	COL4A3	NC_000002.11:g.228162399G>A	NM_000091.4:c.3575G>A	NP_000082.2:p.Gly1192Glu	4				4	Sí	Desconocido	Sí	Desconocido	[29]
240	Mujer	COL4A4	NC_000002.11:g.228009262_228009267delGAGTAT	NM_000092.4:c.81_86delACTCAT	NP_000083.3:p.Leu28_Ile29del	9				9	Sí	Desconocido	No	Desconocido	[29]
241	Hombre	COL4A4	NC_000002.11:g.227973556C>T	NM_000092.4:c.686G>A	NP_000083.3:p.Gly229Asp	15				15	Sí	Desconocido	No	Desconocido	[29]
242	Hombre	COL4A4	NC_000002.11:g.227954647C>T	NM_000092.4:c.1396G>A	NP_000083.3:p.Gly466Arg	13				13	Sí	Desconocido	Sí	Desconocido	[29]
243	Hombre	COL4A4	NC_000002.11:g.227946893C>G	NM_000092.4:c.1634G>C	NP_000083.3:p.Gly545Ala	25				25	Sí	Desconocido	Sí	Desconocido	[29]
244	Hombre	COL4A4	NC_000002.11:g.227946893C>G	NM_000092.4:c.1634G>C	NP_000083.3:p.Gly545Ala	5				5	Sí	Desconocido	No	Desconocido	[29]
245	Mujer	COL4A4	NC_000002.11:g.227924333delC	NM_000092.4:c.2171delG	NP_000083.3:p.Arg724Leufs*29	40				40	Sí	Desconocido	No	Desconocido	[29]
246	Hombre	COL4A4	NC_000002.11:g.227915821C>T	NM_000092.4:c.3022G>A	NP_000083.3:p.Gly1008Arg	8				8	Sí	Desconocido	No	Desconocido	[29]
247	Hombre	COL4A4	NC_000002.11:g.227886772_227886773insG	NM_000092.4:c.4208_4209insC	NP_000083.3:p.Gly1404Argfs*29	41				41	Sí	Desconocido	Desconocido	Desconocido	[29]
248	Hombre	COL4A4	NC_000002.11:g.227872069C>T	NM_000092.4:c.5045G>A	NP_000083.3:p.Arg1682Gln	13				13	Sí	Desconocido	No	Desconocido	[29]

249	Hombre	COL4A4	NC_000002.11:g.227872066C>T	NM_000092.4:c.5048G>A	NP_000083.3:p.Cys1683Tyr	14				14	Sí	Desconocido	Sí	Desconocido	[29]
250	Mujer	COL4A3	NC_000002.11:g.228122337G>T	NM_000091.4:c.1006G>T	NP_000082.2:p.Gly336Cys	23	23				Sí	Desconocido	Sí	Desconocido	[29]
251	Mujer	COL4A3	NC_000002.11:g.228131171G>A	NM_000091.4:c.1354G>A	NP_000082.2:p.Gly452Arg	5	5				Sí	Desconocido	Sí	Desconocido	[29]
252	Hombre	COL4A3	NC_000002.11:g.228131171G>A	NM_000091.4:c.1354G>A	NP_000082.2:p.Gly452Arg	2	2				Sí	Desconocido	Sí	Desconocido	[29]
253	Mujer	COL4A3	NC_000002.11:g.228131171G>A	NM_000091.4:c.1354G>A	NP_000082.2:p.Gly452Arg	14	14				Sí	Desconocido	Desconocido	Desconocido	[29]
254	Mujer	COL4A3	NC_000002.11:g.228131171G>A	NM_000091.4:c.1354G>A	NP_000082.2:p.Gly452Arg	39	39				Sí	Desconocido	Desconocido	Desconocido	[29]
255	Hombre	COL4A3	NC_000002.11:g.228131171G>A	NM_000091.4:c.1354G>A	NP_000082.2:p.Gly452Arg	42	42				Sí	Desconocido	No	Desconocido	[29]
256	Mujer	COL4A3	NC_000002.11:g.228131171G>A	NM_000091.4:c.1354G>A	NP_000082.2:p.Gly452Arg	73	73		ESRD		Sí	Desconocido	Sí	Desconocido	[29]
257	Mujer	COL4A3	NC_000002.11:g.228142227G>A	NM_000091.4:c.2083G>A	NP_000082.2:p.Gly695Arg	43	43	38	CRD		Sí	Desconocido	Sí	Sí	[29]
258	Hombre	COL4A3	NC_000002.11:g.228142227G>A	NM_000091.4:c.2083G>A	NP_000082.2:p.Gly695Arg	21	21				Sí	Desconocido	Sí	Desconocido	[29]
259	Mujer	COL4A3	NC_000002.11:g.228142227G>A	NM_000091.4:c.2083G>A	NP_000082.2:p.Gly695Arg	23	23				Sí	Desconocido	Sí	Desconocido	[29]
260	Mujer	COL4A3	NC_000002.11:g.228142227G>A	NM_000091.4:c.2083G>A	NP_000082.2:p.Gly695Arg	40	40				Sí	Desconocido	Sí	Sí	[29]
261	Mujer	COL4A3	NC_000002.11:g.228142227G>A	NM_000091.4:c.2083G>A	NP_000082.2:p.Gly695Arg	41	41				Sí	Desconocido	No	Desconocido	[29]
262	Hombre	COL4A3	NC_000002.11:g.228142227G>A	NM_000091.4:c.2083G>A	NP_000082.2:p.Gly695Arg	42	42				Sí	Desconocido	No	Desconocido	[29]
263	Hombre	COL4A3	NC_000002.11:g.228142227G>A	NM_000091.4:c.2083G>A	NP_000082.2:p.Gly695Arg	43	43				Sí	Desconocido	Sí	Desconocido	[29]
264	Hombre	COL4A3	NC_000002.11:g.228142227G>A	NM_000091.4:c.2083G>A	NP_000082.2:p.Gly695Arg	44	44				Sí	Desconocido	Sí	Desconocido	[29]
265	Mujer	COL4A3	NC_000002.11:g.228142227G>A	NM_000091.4:c.2083G>A	NP_000082.2:p.Gly695Arg		70	70	ESRD		Sí	Desconocido	Sí	Desconocido	[29]
266	Hombre	COL4A3	NC_000002.11:g.228142227G>A	NM_000091.4:c.2083G>A	NP_000082.2:p.Gly695Arg	71	71				Sí	Desconocido	Sí	Desconocido	[29]
267	Mujer	COL4A3	NC_000002.11:g.228157935G>C	NM_000091.4:c.3239G>C	NP_000082.2:p.Gly1080Ala	50	50				Sí	Desconocido	No	Sí	[29]
268	Mujer	COL4A3	NC_000002.11:g.228157935G>C	NM_000091.4:c.3239G>C	NP_000082.2:p.Gly1080Ala		80	72	ESRD		Sí	Desconocido	Sí	Sí	[29]
269	Mujer	COL4A4	NC_000002.11:g.227985840G>A	NM_000092.4:c.217C>T	NP_000083.3:p.Gln73*	7	7				Sí	Desconocido	Sí	Desconocido	[26]
270	Mujer	COL4A4	NC_000002.11:g.227985840G>A	NM_000092.4:c.217C>T	NP_000083.3:p.Gln73*	39	39				Sí	Desconocido	No	Desconocido	[26]
271	Hombre	COL4A4	NC_000002.11:g.227985840G>A	NM_000092.4:c.217C>T	NP_000083.3:p.Gln73*	41	41				Sí	Desconocido	Desconocido	Desconocido	[26]
272	Hombre	COL4A4	NC_000002.11:g.227985840G>A	NM_000092.4:c.217C>T	NP_000083.3:p.Gln73*		65	65	ESRD		Sí	Desconocido	Sí	Desconocido	[26]
273	Hombre	COL4A4	NC_000002.11:g.227985840G>A	NM_000092.4:c.217C>T	NP_000083.3:p.Gln73*		65	65	ESRD		Sí	Desconocido	Sí	Desconocido	[26]
274	Hombre	COL4A4	NC_000002.11:g.227985840G>A	NM_000092.4:c.217C>T	NP_000083.3:p.Gln73*		70	70	ESRD		Sí	Desconocido	Sí	Desconocido	[26]
275	Hombre	COL4A4	NC_000002.11:g.227985840G>A	NM_000092.4:c.217C>T	NP_000083.3:p.Gln73*	75	75	60	ESRD		Sí	Desconocido	Desconocido	Desconocido	[26]
276	Hombre	COL4A4	NC_000002.11:g.227963505C>T	NM_000092.4:c.1109G>A	NP_000083.3:p.Gly370Glu	14	14				Sí	Desconocido	Sí	Desconocido	[26]
277	Mujer	COL4A4	NC_000002.11:g.227963505C>T	NM_000092.4:c.1109G>A	NP_000083.3:p.Gly370Glu	7	7				Sí	Desconocido	No	Desconocido	[26]
278	Hombre	COL4A4	NC_000002.11:g.227963505C>T	NM_000092.4:c.1109G>A	NP_000083.3:p.Gly370Glu	8	8				Sí	Desconocido	No	Desconocido	[26]
279	Mujer	COL4A4	NC_000002.11:g.227963505C>T	NM_000092.4:c.1109G>A	NP_000083.3:p.Gly370Glu	11	11				Sí	Desconocido	No	Desconocido	[26]
280	Hombre	COL4A4	NC_000002.11:g.227963505C>T	NM_000092.4:c.1109G>A	NP_000083.3:p.Gly370Glu	33	33				Sí	Desconocido	No	Desconocido	[26]
281	Mujer	COL4A4	NC_000002.11:g.227963505C>T	NM_000092.4:c.1109G>A	NP_000083.3:p.Gly370Glu	39	39				Sí	Desconocido	Sí	Sí	[26]
282	Mujer	COL4A4	NC_000002.11:g.227963505C>T	NM_000092.4:c.1109G>A	NP_000083.3:p.Gly370Glu	39	39				Sí	Desconocido	Sí	Sí	[26]
283	Mujer	COL4A4	NC_000002.11:g.227963505C>T	NM_000092.4:c.1109G>A	NP_000083.3:p.Gly370Glu	62	62				Sí	Desconocido	Sí	Sí	[26]
284	Hombre	COL4A4	NC_000002.11:g.227963505C>T	NM_000092.4:c.1109G>A	NP_000083.3:p.Gly370Glu		64	53	ESRD		Sí	Desconocido	Sí	Sí	[26]
285	Hombre	COL4A4	NC_000002.11:g.227963505C>T	NM_000092.4:c.1109G>A	NP_000083.3:p.Gly370Glu		68	43	ESRD		Desconocido	Desconocido	Sí	Sí	[26]
286	Hombre	COL4A4	NC_000002.11:g.227963505C>T	NM_000092.4:c.1109G>A	NP_000083.3:p.Gly370Glu		74	59	ESRD		Desconocido	Desconocido	Sí	Sí	[26]
287	Mujer	COL4A4	NC_000002.11:g.227945167C>T	NM_000092.4:c.1795G>A	NP_000083.3:p.Gly599Arg	33	33				Sí	Desconocido	Sí	Desconocido	[26]
288	Hombre	COL4A4	NC_000002.11:g.227945167C>T	NM_000092.4:c.1795G>A	NP_000083.3:p.Gly599Arg	21	21				Sí	Desconocido	No	Desconocido	[26]
289	Mujer	COL4A4	NC_000002.11:g.227945167C>T	NM_000092.4:c.1795G>A	NP_000083.3:p.Gly599Arg	33	33				Sí	Desconocido	No	Desconocido	[26]
290	Mujer	COL4A4	NC_000002.11:g.227945167C>T	NM_000092.4:c.1795G>A	NP_000083.3:p.Gly599Arg	59	59				Sí	Desconocido	No	Desconocido	[26]

291	Mujer	COL4A4	NC_000002.11:g.227945167C>T	NM_000092.4:c.1795G>A	NP_000083.3:p.Gly599Arg		74	74	renal trasplant		Sí	Desconocido	No	Desconocido	[26]
292	Hombre	COL4A4	NC_000002.11:g.227920804C>G	NM_000092.4:c.2573G>C	NP_000083.3:p.Gly858Ala	8	8				Sí	Desconocido	Sí	Desconocido	[26]
293	Mujer	COL4A4	NC_000002.11:g.227920804C>G	NM_000092.4:c.2573G>C	NP_000083.3:p.Gly858Ala	39	39				Sí	Desconocido	No	Desconocido	[26]
294	Hombre	COL4A4	NC_000002.11:g.227919418C>T	NM_000092.4:c.2752G>A	NP_000083.3:p.Gly918Arg	13	13				Sí	Desconocido	Sí	Desconocido	[26]
295	Hombre	COL4A4	NC_000002.11:g.227919418C>T	NM_000092.4:c.2752G>A	NP_000083.3:p.Gly918Arg	18	18				Sí	Desconocido	No	Desconocido	[26]
296	Hombre	COL4A4	NC_000002.11:g.227919418C>T	NM_000092.4:c.2752G>A	NP_000083.3:p.Gly918Arg	40	40				Sí	Desconocido	Sí	Desconocido	[26]
297	Hombre	COL4A4	NC_000002.11:g.227919418C>T	NM_000092.4:c.2752G>A	NP_000083.3:p.Gly918Arg		66	54	ESRD		Sí	Desconocido	Sí	Desconocido	[26]
298	Mujer	COL4A4	NC_000002.11:g.227886884delG	NM_000092.4:c.4097delC	NP_000083.3:p.Pro1366Argfs*22	6	6				Sí	Desconocido	Sí	Desconocido	[26]
299	Hombre	COL4A4	NC_000002.11:g.227886884delG	NM_000092.4:c.4097delC	NP_000083.3:p.Pro1366Argfs*22	44	44				Sí	Desconocido	No	Desconocido	[26]
300	Mujer	COL4A4	NC_000002.11:g.227875157C>T	NM_000092.4:c.4394G>A	NP_000083.3:p.Gly1465Asp	37	37				Sí	Desconocido	Sí	Desconocido	[26]
301	Hombre	COL4A4	NC_000002.11:g.227875157C>T	NM_000092.4:c.4394G>A	NP_000083.3:p.Gly1465Asp	34	34				Sí	Desconocido	No	Desconocido	[26]
302	Mujer	COL4A4	NC_000002.11:g.227875157C>T	NM_000092.4:c.4394G>A	NP_000083.3:p.Gly1465Asp	55	55				Sí	Desconocido	No	Desconocido	[26]
303	Mujer	COL4A4	NC_000002.11:g.227875157C>T	NM_000092.4:c.4394G>A	NP_000083.3:p.Gly1465Asp	75	75				Sí	Desconocido	No	Desconocido	[26]
304	Mujer	COL4A4	NC_000002.11:g.227875157C>T	NM_000092.4:c.4394G>A	NP_000083.3:p.Gly1465Asp		80	80	ESRD		Sí	Desconocido	Desconocido	Desconocido	[26]
305	Hombre	COL4A4	NC_000002.11:g.227875111delG	NM_000092.4:c.4444delC	NP_000083.3:p.Leu1482Trpfs*70	12	12				Sí	Desconocido	No	Desconocido	[26]
306	Hombre	COL4A4	NC_000002.11:g.227875111delG	NM_000092.4:c.4444delC	NP_000083.3:p.Leu1482Trpfs*70		31				Sí	Desconocido	Sí	Desconocido	[26]
307	Hombre	COL4A4	NC_000002.11:g.227875111delG	NM_000092.4:c.4444delC	NP_000083.3:p.Leu1482Trpfs*70	44	44	38	ESRD		Desconocido	Desconocido	Desconocido	Sí	[26]
308	Hombre	COL4A4	NC_000002.11:g.227875111delG	NM_000092.4:c.4444delC	NP_000083.3:p.Leu1482Trpfs*70	46	46				Sí	Desconocido	No	Sí	[26]
309	Hombre	COL4A4	NC_000002.11:g.227872792_227872794delGAC	NM_000092.4:c.4749_4751delGTC	NP_000083.3:p.Gln1583_Ser1584delinsHis	48	48				Sí	Desconocido	Sí	Sí	[26]
310	Hombre	COL4A4	NC_000002.11:g.227872792_227872794delGAC	NM_000092.4:c.4749_4751delGTC	NP_000083.3:p.Gln1583_Ser1584delinsHis	16	16				Sí	Desconocido	No	Desconocido	[26]
311	Hombre	COL4A4	NC_000002.11:g.227872792_227872794delGAC	NM_000092.4:c.4749_4751delGTC	NP_000083.3:p.Gln1583_Ser1584delinsHis		56	52	ESRD		Sí	Desconocido	Sí	Desconocido	[26]
312	Hombre	COL4A4	NC_000002.11:g.227872792_227872794delGAC	NM_000092.4:c.4749_4751delGTC	NP_000083.3:p.Gln1583_Ser1584delinsHis	57	57				Sí	Desconocido	No	Desconocido	[26]
313	Hombre	COL4A3	NC_000002.11:g.228167817G>A	NM_000091.4:c.3946G>A	NP_000082.2:p.Gly1316Ser	12	12			12	Desconocido	Desconocido	Sí	Desconocido	[26]
314	Hombre	COL4A3	NC_000002.11:g.228124575G>A	NM_000091.4:c.1096G>A	NP_000082.2:p.Gly366Arg	18	18			18	-	-	Sí	-	This Study
315	Mujer	COL4A3	NC_000002.11:g.228121068G>A	NM_000091.4:c.943G>A	NP_000082.2:p.Gly315Ser	12	12			12	-	Sí	-	-	This Study
316	Mujer	COL4A3	NC_000002.11:g.228121068G>A	NM_000091.4:c.943G>A	NP_000082.2:p.Gly315Ser	50	50			50	-	Sí	-	-	This Study
317	Hombre	COL4A3	NC_000002.11:g.228121068G>A	NM_000091.4:c.943G>A	NP_000082.2:p.Gly315Ser	70	84	70	Dialysis	70	-	-	-	Sí	This study
